# Some Facts in Regard to Private Medical Practice in New Orleans

**Published:** 1874-02-01

**Authors:** 

**Affiliations:** New Orleans


### SOME FACTS IN REGARD TO PRIVATE MEDICAL PRACTICE IN NEW ORLEANS.

TO THE EDITORS OF THE MEDICAL EXAMINER—*My Dear Sirs:*—After some deliberation, I have come to the conclusion that some facts in regard to private medical practice in New Orleans will prove interesting to your readers I have, therefore, determined that this shall be the subject for my first letter, reserving for future letters the discussion of hospitals and hospital experiences, medical institutions, societies, and other matters of general interest to our profession.

As in all large cities, a group of statistics which only represents the results of observations personal to a single practitioner, must be understood to possess but small claim to our confidence as exhibiting such results as would be shown if the aggregate statistics of the city could be studied. They also differ, even yet more widely, from the statistics gathered under the personal observation of other individual practitioners, according as the circumstances surrounding each may determine different results. It becomes, therefore, necessary for me to say, in the beginning of my letter, that the surroundings of my patients are as favorable for successful results as can be obtained in any part of this city. My practice is almost wholly confined to the fourth, or "garden," district of the city, and yet is sufficiently central to afford very nearly, if not quite, as great a degree of exemption from the influence of swamp-poison as is found in the diseases of the most thickly populated portions of the town. With scarcely an exception, my patrons are in circumstances of life which enable them to procure everything necessary to the recovery and comfort of the sick. I am called at the earliest moment after development of attacks of sickness, and feel altogether unrestrained in regard to the amount of service I am to render to my patients. I am, therefore, governed on this point solely by my own ideas of expediency and propriety.

From the 1st of January, 1873, to date, I have prescribed for over eleven hundred patients in private practice. The exact enumeration on my visiting-list is nine hundred and forty-two; but, as this list is only kept for my convenience in rating my fees, it is far from representing the actual number of patients prescribed for. In all instances where more than one member of a family were sick at the same time, the name of the head of the family, and one number, were used to indicate all the services rendered. During the summer and autumn, dengue was epidemic; and, in many instances, whole families would be ill at the same time; I am, therefore, quite sure that eleven hundred is a reasonable estimate of the number of patients treated.

If we admit the correctness of this latter estimate, the deaths have been precisely one per cent. I wish now to call attention more particularly to these eleven deaths, with especial respect to their causation.

On January 30th, a girl aged eighteen years, who had visited the city for the purpose of participating in the festivities usual to that season, was suddenly seized with a chill. For the twenty-four hours ensuing, she refused to allow her friends to call in medical aid, assuring them that it was but an ordinary chill, returns of which she had frequently suffered in the intensely malarial region where she resided, and which she was in the habit of treating with domestic remedies. On the afternoon of the 31st, I was asked to see her. The first glance at the patient revealed the nature of her disease. An insufferable cephalalgia provoked incessant moans; the head was drawn backwards, and the hyperæsthesia—especially of the legs—caused her to scream with pain at every attempt to move them. The first steps of treatment looked to the subtraction of the malarial complication. Quinine, in ten grain doses, was given every fourth hour. At the same intervals, but at different hours, one-half grain of morphia, and one-fourth grain of extract of belladonna were given. The remedies were continued through the night; but neither sleep, nor respite from pain, was procured. On the morning of the 1st of February, one of the most skillful practitioners was requested to assist me in the further treatment of the case. This gentleman had just treated one or more cases of cerebro-spinal meningitis with chloral hydrate, and was quite pleased with its effects.

Accordingly, it was agreed to give the patient fifteen grains every half hour, until sleep resulted. Immediately after the third dose had been exhibited, we returned, in order to observe its effects. The patient expressed herself as being freer from pain than at any time since her attack. While very carefully noting her symptoms, we detected a slight intermittency of the pulse, with an occasional pause between the acts of respiration. Although these symptoms were so little marked that they might readily have escaped an investigation less critical than that which was instituted, they still clearly conveyed to my mind indications of commencing effusion. I awoke the patient from a slumber into which she had, for the first time, fallen, in order to determine if she was becoming comatose. She replied to my inquiries without hesitation, stating that she felt better; was sure that she could sleep; and that on my morning visit I would find her better. Within the following hour her friends observed that her breathing was less audible, and had hardly time to gather around her bed to witness a death as quiet as it was instantaneous. Was this death due to a sudden drowning of vital tracts of the cord, by a rapid effusion? or, did the chloral have anything to do in determining it?

This is the only case of cerebro-spinal meningitis which has occurred in my private practice during the present year.

The second death occurred, also, on the 1st of February, from tubercular meningitis. It was in the case of a female child, aged twenty-two months, and had been brought here from Iowa by its parents, one of whom had been ordered to a Southern climate on account of tubercular disease.

The third death occurred on the 25th of February, in the person of a public official, from chronic dysentery. This man was sixty-three years old, and had led a very irregular life. Although, as you are aware, chronic dysentery is far more common in this climate than yours, my experience has brought me to believe that, in this city at least, it is rare, except as resulting from uncured acute attacks, occurring in the country, or from excessive use of alcoholic drinks.

The fourth death took place on the 21st of June; a man aged sixty-five; of chronic gastritis, of long standing.

The fifth and sixth deaths took place on September 3d and 28th, and were returned as having been due to congestion. Both were children between four and five years of age. The first was the son of a physician. He began to complain on the second day of September, with symptoms which were supposed to denote gastric, or intestinal derangement. On the 3d of September I saw him in the forenoon, and found him somnolent and lethargic. When aroused, he replied to questions, and took the drinks which were offered him, swallowing them without difficulty, relapsing immediately into a state of quiet stupor In the early part of the night, one or two convulsions occurred, and death speedily followed, preceded by complete coma. I presume that such a clinical history in your latitude would be interpreted to denote effusion, either from a tubercular or syphilitic diathesis, or from traumatic causes. Here, we have constantly to keep in view the fact that our atmosphere is liable to become the medium of transmission of certain specific poisons, capable of producing "congestions," with all their varied phenomena. The parents, and other children of this family, are perfectly free from any diathetic taint, and so, also, no doubt, was the little patient himself. He had spent the previous summer in the country, where he was exposed to malaria. Then, again, within the ten days prior to his death, a fatal case of yellow fever had occurred, on the same street, and but a few doors from his residence. While we admit the fact that it is often impossible to assert a differential diagnosis between some cases of malarial fever, and some cases of yellow fever, previous to death, there are *post-mortem* changes of surface here so uniformly taking place in yellow fever, that we generally hold that death is due to some other cause, if the body does not become yellow after death. This *post-mortem* change did not take place with the little child.

The next death was in the person of a merchant, sixty-two years of age, whose heavy pecuniary losses and misfortunes had harassed him to an unusual degree. He awoke at three o'clock at night, complaining of great difficulty of breathing. I saw him at half past three, and found him propped in a sitting posture on the bed, with a scarcely perceptible pulse, cold surface, and a very rapid accumulation of secretion in the bronchial tubes. Death occurred in less than two hours. In making an official return of the death, I ascribed it to cardiac angina, leaving the question of structural disease unmentioned. Unless some evidence of organic disease is otherwise afforded, death from angina is not strictly positive proof of its existence.

The eighth death was from aneurism of the descending aorta, with pressure upon the left bronchus. A few slight hæmorrhages occurred about a month before death, which, however, were due to asthenia from continual pain and interruption of the functions of the lungs and heart. The subject was a merchant, fifty-nine years of age.

The next fatal case was one of chronic diarrhœa, in the person of a merchant, fifty-five years old, who had lived intemperately for some years of his life.

Then, on the 6th of December, a German, aged sixty years, died of cirrhosis of the liver. He died comatose, without any interruption of function, in any emunctory, sufficient to justify a diagnosis of the cause of the coma. Is there a condition of blood-poisoning entitled to the designation of cholesteræmia? If so, does it possess symptoms so distinctive that the disease may possess a clinical history, and prove capable of diagnosis?

The eleventh death took place on the 7th of December, and was due to phthisis pulmonalis. This patient was a worthy and well-to-do negress, aged fifty years. This is certainly not a good climate for consumptives. Perhaps, we may say, with Bowditch, that it is too low—too close both to a river-bed and to the sea-level. But, while I say, quite positively, that the climate is not a good one for consumptives—meaning by this that they do not do well when the disease is already developed—further observation is required to satisfy me that the disease originates here in a ratio to other diseases, as great as is observable at the North.

I have now given you, very frankly, the fatal cases of a year's practice. If it should meet with your approval, I will, in my next, give you an equally candid account of my successes, more especially in acute cases, as, for example, pneumonia, and the essential fevers.

MEDICUS MERIDIONALIS.

New Orleans, Dec. 28th, 1873.

